# Effect of *Rubus idaeus L.* Consumption During Pregnancy on Maternal Mice and Their Offspring

**DOI:** 10.1089/jmf.2021.0078

**Published:** 2022-02-11

**Authors:** Marie Hastings-Tolsma, Ryan T. Stoffel, Alexandra S. Quintana, Robert R. Kane, Jacob Turner, Xuan Wang

**Affiliations:** ^1^ Louise Herrington School of Nursing, Baylor University, Dallas, Texas, USA.; ^2^ Department of Nursing, University of Johannesburg, Johannesburg, South Africa.; ^3^ Animal Program Director & Attending Veterinarian, Baylor University, Waco, Texas, USA.; ^4^ Department of Chemistry and Biochemistry, Baylor University, Waco, Texas, USA.; ^5^ Director, Institute of Biomedical Studies, Baylor University, Waco, Texas, USA.; ^6^ Department of Mathematics & Statistics, Stephen F. Austin State University, Nacogdoches, Texas, USA.; ^7^ Biostatistician II, Baylor Scott & White Research Institute, Dallas, Texas, USA.

**Keywords:** behavioral assessment, gestation length, mice, mice offspring, pregnancy, Rubus idaeus L.

## Abstract

The trigger for human labor is a scientific mystery. This research examined *Rubus idaeus* (RI), commonly referred to as red raspberry, which is widely purported to be efficacious in promoting parturition processes and favorable birth outcomes. This randomized controlled trial sought to determine the influence of RI consumption during gestation on C57BL/6N Tac mice and their offspring. The aims of this study were to (1) determine differences in the length of gestation, gestational weight gain, and litter size where RI is consumed daily at varied strengths and (2) determine differences in offspring characteristics and behavior where maternal RI consumption occurred. Once paired, mice were randomly assigned to one of three groups: placebo (*n* = 10) receiving plain water, RI aqueous extract fluid of 1.78 mg/mL (*n* = 10), or RI aqueous extract fluid of 2.66 mg/mL (*n* = 10). All received the same standardized diet throughout gestation. Pregnant mice were weighed with chow intake and fluid consumption determined daily. Gestation length and litter size were recorded at the time of birth. Differences in offspring characteristics were also determined and included physical characteristics (weight, physical development) and neuromotor reflexes and behaviors (locomotive abilities, geotaxis reflex, cliff avoidance reflex, and swimming development). When compared with controls, high-dose RI ingestion resulted in shorter length of gestation and smaller litter size (*P* ≤ .05). There was also an increase in fluid consumption and a decrease in pup weights on postnatal day 4 and 5 with RI treatment (*P* ≤ .05). Altogether, results suggest that RI influences parturition and fecundity processes with transplacental exposure impacting offspring characteristics.

## INTRODUCTION

Human labor is both complex and highly regulated involving coordination between key reproductive tissues. While the exact mechanism is unknown, the cervix and myometrium are remodeled through the gestational process in preparation for birth.^1–4^ Successful remodeling through architectural change is of significance as it influences the physiological onset and progression of labor.^5,6^

The hunt for safe and efficacious compounds to accomplish birth has received widespread attention but has been met with little success. With limited drug development targeting labor processes,^7^ study of botanicals may demonstrate therapeutic efficacy. One botanical that has received relatively little attention is *Rubus idaeus* (RI; *Rubus idaeus L*, family Rosaceae)—a powerful antioxidant^8^ more commonly known as red raspberry. RI is widely purported to be efficacious in promoting cervical remodeling, stimulating labor, and improving perinatal outcomes.^9,10^ Globally, RI is used by as many as a third of all pregnant women,^11^ with a prevalence of 7–58% worldwide.^12,13^ Typically recommended as a daily tea (one to three cups per day) during the last 4–6 weeks of gestation, little is known about the impact of RI in inducing parturition activity or its safety and efficacy in humans.^10,14^

RI mechanism of action has been studied in select animal models,^15–17^ demonstrating evidence for use as a uterotonic.^18^ While the effectiveness of ingested RI on *gestation, labor, and birth processes* is unknown, the bioactive components in RI extract have been identified. RI is known to contain many phytochemicals, which are primarily derivatives of alkaloids, phenolics, terpenoids, and glycosides.^19,20^ Flavonoid compounds are the primary RI phenolics,^21^ with epigallocatechin gallate (EGCG), kaempferol, and quercetin of particular interest as they are capable of binding to a variety of enzymes inhibiting their activity.^22^ RI is anthocyanin-rich, containing as many as eight anthocyanins.^23,24^ Kaempferol has been found to agonize several signaling pathways known to promote cell apoptosis,^25^ and quercetin has high bioavailability and promotes inflammatory gene expression.^26^ Furthermore, quercetin has been found to interfere in development of the endometrium during the peri-implantation period^27^ and has been found to decrease cell proliferation and induce cell death.^28^ *Catechins*, antioxidants found in tea, scavenge oxidants before cell injuries occur. Two major catechins, gallic acid and ellagic acid,^29^ and specifically sanguiin H-6 and lambertiannin C, have vasodilation activity.^30^ Consistent with theory, RI use in pregnancy likely stimulates proinflammatory cytokine activity, vasodilation, and apoptosis of cervical and myometrial cells, likely from flavonoids and catechins. These bioactive compounds have the potential to play a key role in triggering parturition processes.

The purpose of this research was to understand how RI effected parturition and offspring development. Examination of the effect of RI in humans is clinically and ethically challenging, making use of animal models important. While parturition processes are known to be species-specific,^31^ mice DNA organization and gene expression is similar to humans with a comparable reproductive system,^32^ thus making a murine model ideal.

The aims of this study were to (1) determine differences in the length of gestation, gestational weight gain including food and fluid consumption, and litter size where RI was consumed daily at varied strengths and (2) determine differences in offspring characteristics and behavior.

## MATERIALS AND METHODS

### Plant material

RI was used at two standardized aqueous concentrations, testing against the placebo, to determine the influence on length of gestation and other perinatal outcomes in a mouse model.

### Quality of source material (RI)

Voucher specimen RI material was supplied by a certified organic manufacturer, Traditional Medicinal^®^. The product was a homogeneous dried botanical packaged as raw product, shipped overnight, and placed in cool dry storage. This handling minimized variability in the amount of product delivered.

### Extract preparation

To produce RI extract, 53.95 g of RI was added to 500 mL of deionized (DI) boiling water. These quantities were chosen after pilot experimentation to efficiently and uniformly afford the quantity of extract needed for the course of experimentation. The extract preparation and DI water were gently boiled for 15 min. The solution was then cooled while vigorously stirring for 1 h. The remaining solution was filtered, yielding ∼400 mL of tea extract solution, which was evenly divided into four Erlenmeyer flasks and frozen by rotating slowly in a −78°C dry ice–acetone bath. Flasks were then placed on a lyophilizer and left to sublimate over 3 days, yielding 10.4 g of dried tea extract. The extract was sealed and stored at 4°C until needed. To deliver the two concentrations, extract was dissolved in DI water to yield concentrations of 2.66 mg/mL (high dose) and 1.78 mg/mL (low dose). These concentrations were calculated to deliver doses scaled to the mouse with an equivalence to human consumption of one or two cups of tea—the typical recommended daily amount.

### Mice

C57BL/6N Tac mice (30 males and 30 females) were received from Taconic Biosciences (Rensselaer, NY, USA) at 8 weeks and placed in a specific pathogen-free environment. C57BL/6N Tac mice are a common inbred mouse model and are a preferred model for studying diet-induced experiments. These mice are genetically identical within a generation and across generations; their use permits reproducibility and consistency across experiments and allows conclusions to be drawn in the absence of genetic variation considerations.^33^

For C57BL/6N Tac mice, the mean number of days to litter is 21 and the average litter size is six to eight pups.^34^ Mice were acclimated for 1 week in an environment with controlled ambient temperature (22–25°C), room humidity (30–70%), and a standardized 12:12 h light cycle. The housing room had ≥10 air changes per hour. Female mice were then bred, and a vaginal copulatory plug in the morning was taken as evidence of successful mating and was considered gestational day 1. Following mating, female mice were moved to individual cages.

Mice were randomly assigned to one of three fluids with *ad libitum* access throughout gestation. A standard water bottle for fluids contained either DI water (*n* = 10) or one of two RI aqueous extract fluids: 1.78 mg/mL (*n* = 10) or 2.66 mg/mL (*n* = 10), with bottles weighed daily. Weighing bottles containing the standardized low- and high-dose extract fluids each day allowed for determination of the approximate amount of extract consumed by each mouse. The standard vivarium rodent diet (LabDiet^®^ 5V5R) was used throughout gestation and the postnatal period. The amount of chow ingested was determined by weighing the food container when filled and again each day. All mice were placed on facility water following birth.

### Sample size

The resource equation method^35^ was used to calculate sample size with 27 dams needed for adequate study power. Use of 10 mice per group allowed for expected attrition, anticipated to be less than 10%.

### Scale

The Ohaus^®^–SPX2201 scale was used to obtain maternal and pup weights and to weigh food containers and fluid bottles. The digital scale had a capacity of 2200 g, with a pan size of 5.5″ × 6.7″ and accuracy within 0.1 g.

### Maternal outcomes measures: length of gestation, gestational weight gain, and litter size

Dams were caged individually with their litter. *Length of gestation* was measured in days from the time that a copulatory plug was noted until birth occurred. Mice generally give birth at night, but exact time of birth was undetermined as remote facility monitoring was unavailable. Rather, mice cages were checked twice each day to observe for birth. Where birth occurred more than 12 h before term (21 days), a spontaneous preterm birth was recorded. *Gestational weight gain* was determined by weighing mice at cage placement and again at the same time each day. The amount of fluid (mL/day) and chow (g/day) ingested were recorded daily across gestation. *Litter size* was determined by pup count at the time of birth (viable and nonviable).

### Pup outcome measures: physical characteristics, neuromotor reflex, and behavior

Measurements of physical characteristics and behavior of offspring were done between 8 and 11 a.m. beginning on postnatal day (PND) 4, with outcome measures determined on specific postnatal days until day 14. Mice typically begin eating solid food between PND 14–16; weaning typically occurs between 21 and 28 days. Determination of offspring characteristics and behavior was not begun until PND4 to minimize disruption of maternal behaviors (retrieval, licking, and nursing). Early disruption of the mother, pup, nest, and cage is known to influence mother–pup interactions.^36^

The presence and development of several *physical characteristics* were determined at specific time points, with day of birth as PND00. Pup weights were determined in the morning on PND4, 5, 6, 7, 8, 10, 12, and 14. Body development was evaluated to determine the presence or absence of hair appearance (PND4, 6, 7, 8, 10, 12, 14), and low incisor eruption and bilateral eye opening (PND4, 6, 8, 10, 12, 14).

Pup locomotive abilities and general body strength were evaluated by scoring *righting reflex* ability on PND4, 5, 6, and 7. This reflex involves the ability of the pup to return to four paws after having been placed in a supine position (maximum 30 seconds). The *geotaxis reflex*, or the orienting response and movement expressed in opposition to curves of a gravitational vector, was determined by placement on a 45° inclined plane on PND8. A negative geotaxis reflex was determined if the pup turned toward the high end of the plane, moving uphill (maximum 120 seconds). *Cliff avoidance reflex* was determined at PND10 with pups placed on a wooden platform raised 20 cm from a tabletop. The forepaws and snout of the animal were positioned so that the edge of the platform passes just behind an imaginary line drawn between the eye orbits. The amount of time required for a complete retraction of the head behind the border of the platform was recorded (maximum 120 seconds). *Swimming development* was determined on PND12 with each pup individually placed in a tank of water (28°C, 10 seconds) with direction, angle in the water (head position), and limb usage observed. Direction scores consisted of head position (0–2), front limb (0–2), hind leg (0–2), tail movement (0–2), and line of swim (0–2); total possible score was 10. Angle scores consisted of head submerged; nose at the surface (1), nose and top of head at or above the surface but ears still below the surface (2), half of ears above the surface (3), and ears completely above the surface. Limb usage scores consisted of no paddling, paddling with all four limbs (1), and paddling with hind limbs only with forelimbs stationary (2).

The same two trained operators (R.T.S. and A.S.Q.) assessed and obtained all measurements of mice. Separated pups were returned to the cage/mother immediately after observations.

### Statistical analyses

Statistical analyses of the results were evaluated between the control (placebo) and treatment groups. Mean ± standard deviation (SD) was calculated. Unpaired *t*-tests and one- or two-way analysis of variance, followed by the Tukey test or Bonferroni correction (in the case of a normal distribution) or non-parametric Kruskal–Wallis and Mann–Whitney *U* tests (in the case of abnormal distribution) were determined. Assessment of the distribution of the data was evaluated using the Shapiro–Wilk test. SPSS 20.0 Software (SPSS Inc., Chicago, IL, USA) was used for all statistical analyses. A statistically significant difference was indicated with *P* ≤ .05.

## RESULTS

### Length of gestation

**FIG. 1.**
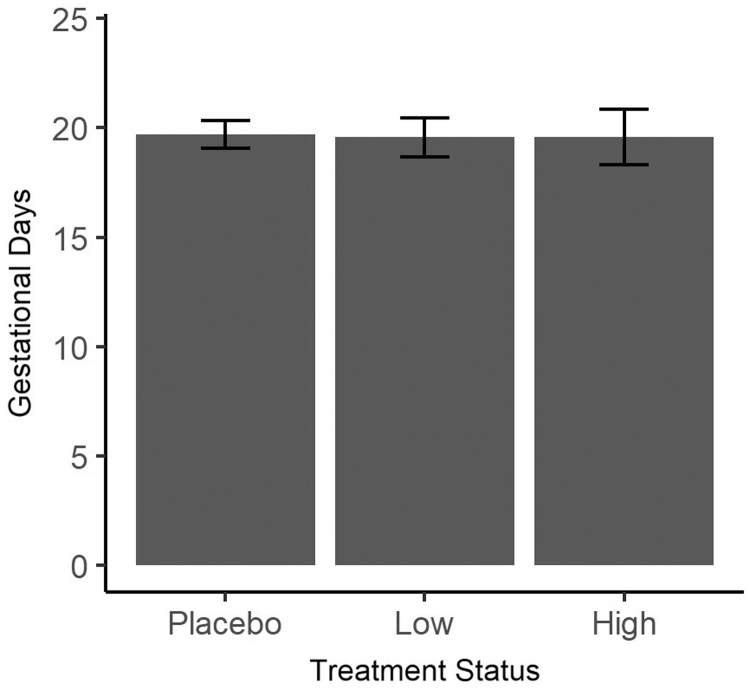
Length of gestation (in days) by *Rubus idaeus* extract treatment group. Concentrations were 2.66 and 1.78 mg/mL for high- and low-dose, respectively. The placebo group received plain water. There is no significant difference between the treatment groups versus the placebo group on gestation days, although there was a trend in indicating shorter gestation in the high-dose group (one-way ANOVA followed by Tukey's multiple comparison correction). ANOVA, analysis of variance.

RI ingestion resulted in differences in length of gestation compared with the placebo. While there were no significant differences in length of gestation when ingesting low dose or placebo, those ingesting high dose tended to have shorter gestation ($\bar{x}$ = 19.57, SD: 1.27, range: 18–22) when compared with the placebo group ($\bar{x}$ = 19.71, SD: 0.64, range: 19–20.5) (*P* ≤ .04) (Fig. 1).

### Gestational weight gain

**FIG. 2.**
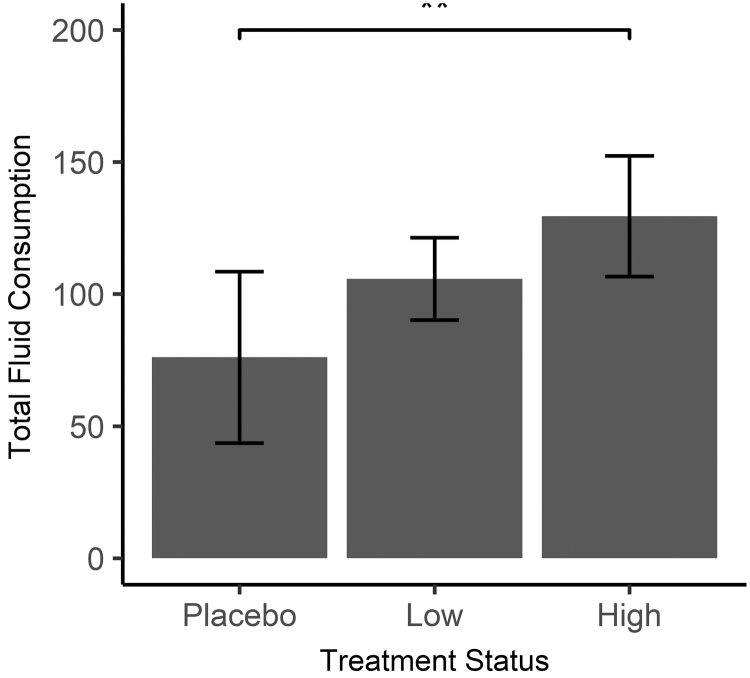
Total fluid consumption (in milliliters) during gestation by *Rubus idaeus* extract treatment status (*P* ≤ .05) (one-way ANOVA followed by Tukey's multiple comparison correction). The high-dose group had significant greater consumption when compared with the placebo (control, plain water) group.

Across groups, total gestational weight gain of dams ranged from 7.7 to 21.6 g. Gestational weight gain for the placebo group ranged from 15.7 to 21.6 g ($\bar{x}$ = 18.74, SD: 1.99), 9.3–19.2 g ($\bar{x}$ = 16, SD: 3.42) in the low-dose group, and 7.7–21.1 g ($\bar{x}$ = 15.37, SD: 4.42) in the high-dose group. There were no significant differences in the total gestational weight gain when comparing groups. When considering food and fluid consumption throughout gestation, food consumption ranged from 24.2 to 102.7 g, with no significant differences noted between groups: placebo ($\bar{x}$ = 71.34, SD: 24.12, range: 24.2–102.7), low dose ($\bar{x}$ = 72.13, SD: 5.72, range: 66–82.1), high dose ($\bar{x}$ = 66.81, SD: 11.19, range: 50.3–79.2). Fluid consumption ranged from 29 to 128 mL across groups with a significant difference noted when comparing high dose ($\bar{x}$ = 129.44, SD: 22.82, range: 108–173.4) with placebo ($\bar{x}$ = 76.04, SD: 32.49, range: 29–128) with consumption greater in the high-dose group (*P* ≤ .05) (Fig. 2).

### Litter size and viability

**FIG. 3.**
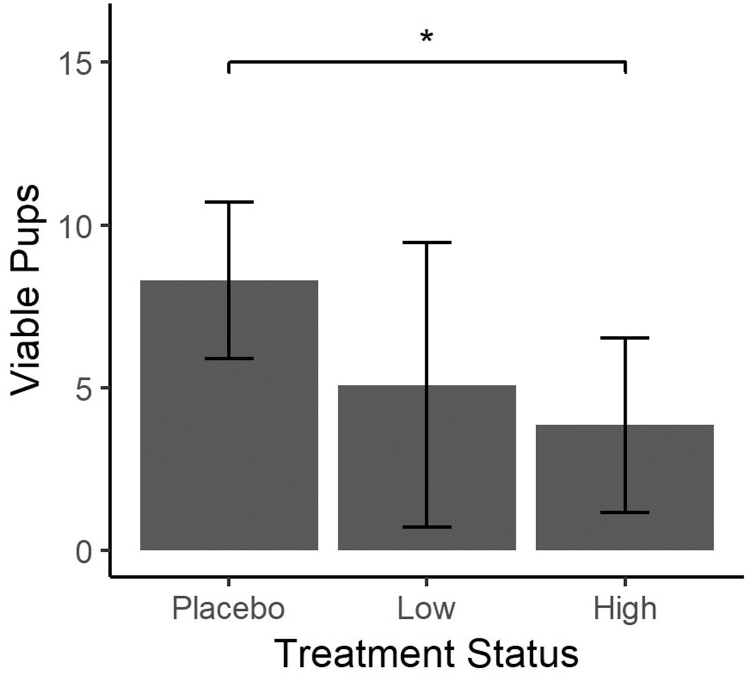
Number of viable pups per litter in dams exposed to *Rubus idaeus* extract throughout entire gestation. Results are presented as mean ± SEM (*P* ≤ .04) versus placebo (control receiving plain water) (one-way ANOVA followed by Tukey's multiple comparison correction). SEM, standard error of the mean.

When comparing litter size (viable and nonviable) between the groups, there were significantly fewer pups with both low- and high-dose RI treatment (*P* ≤ .05). The placebo group had a greater number of pups ($\bar{x}$ = 9.2, SD: 1.69, range: 6–11) than did the low-dose ($\bar{x}$ = 7.1, SD: 3.0, range: 1–10) or high-dose ($\bar{x}$ = 5.8, SD: 3.0, range: 1–8) groups. There were also fewer viable pups in RI-treated groups when compared with the placebo group. The placebo group had an average of 8.3 viable pups (SD: 2.4, range: 3–11), 5.1 (SD: 4.3, range: 0–10) in the low-dose group, and 3.8 (SD: 2.6, range: 0–7) in the high-dose group. Differences between groups were statistically significant (*P* ≤ .04) (Fig. 3).

Dams were moved to an individual cage following pairing and an observed presence of a vaginal copulatory plug. However, for eight dams, despite incremental maternal weight gain during the first week, weight gain leveled off or declined. It is unknown if these mice aborted, but additional pairings were undertaken. Of note, there was no relationship between the number of pairings and litter size. Finally, there were notable differences in the timing and number of pups lost following birth. Nonsurvival of pups following birth demonstrated 22 (range: 3–10) in the control group, and 18 (range: 1–9) and 13 (range: 1–4) in the low- and high-dose groups, respectively. Pups were typically lost at PND2.25 (range: 1–3), PND2.75 (range: 2–5), and PND3.8 (range: 3–8) in the control, low-, and high-dose groups, respectively.

### Pup physical characteristics: body development

**FIG. 4.**
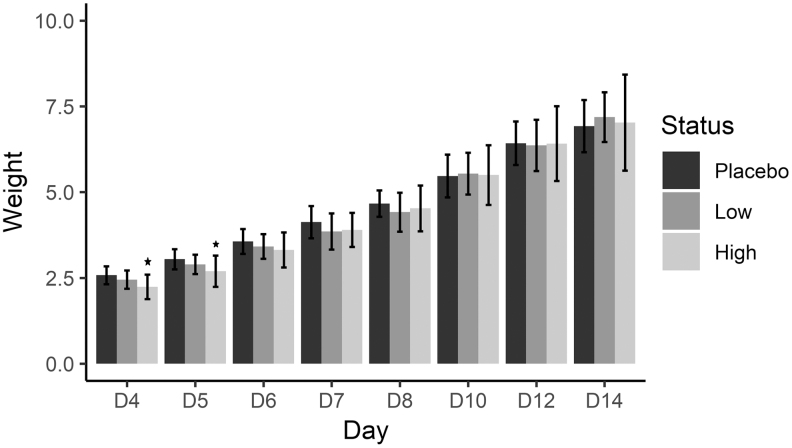
Weight of offspring mice exposed prenatally to *Rubus idaeus* extract. Results are presented as mean ± SEM (*P* ≤ .05) (one-way ANOVA with a random effect to account for repeated observations within the same litter was conducted at each time point followed by Tukey's multiple comparison correction). D, postnatal day.

Body development examined pup weight (PND4, 5, 6, 7, 8, 10, 12, 14), appearance of hair/fur (PND4, 6, 7, 8, 10, 12, 14), and lower incisor eruption and bilateral eye opening (PND4, 6, 8, 10, 12, 14). When comparing groups, there were no significant differences except pup weights, which were lower at PND4 and PND5 when comparing high-dose treatment with low dose and placebo (*P* ≤ .05) (Fig. 4).

### Pup locomotive abilities and general body strength

Righting abilities were not statistically different among groups, although there was a trend for time to righting to increase with higher RI treatment at PND4 and PND6; time to righting incrementally decreased at PND5 and PND7 when compared with placebo. Where examining geotaxis at PND8, there were no significant differences among groups. However, RI treatment tended to increase time when compared with the placebo group. Similarly, cliff avoidance reflex at PND10 found that those in low- and high-dose treatment groups generally required less time as the dose increased, but when compared with placebo, findings were not statistically significant.

General body strength was monitored in swimming development at PND12 by observing swim direction, head position, line of swim, and use of head, paws, and tail movement. Scores (0–2) were assigned for each, and a total score (0–10) was determined. No significant differences were found for each metric or for the overall swim score among groups. There was, however, a trend toward lower scores for head and hind leg position and swim line, as well as a lower overall total swim score, with RI treatment.

## DISCUSSION

This is the first study using a mouse model to evaluate the impact of daily RI consumption on length of gestation and gestational weight gain that measured food and fluid consumption, litter size, and effect on pup behavior and physical development following transplacental exposure.

### Pregnancy and parturition processes

The high-dose RI-treated mice demonstrated significantly shorter gestation. The phytochemicals in RI are primarily derivatives of alkaloids, phenolics, terpenoids, and glycosides.^19,20^ The phenolics are likely the most active biochemicals and include flavonoid compounds such a flavones, isoflavones, flavonones, catechins, and anthocyanins.^21^ The polyphenols epigallocatechin, kaempferol, quercetin, and genistein are of particular interest as they are capable of binding to a variety of enzymes, inhibiting their activity.^22,37,38^

The potential role of RI in parturition is likely related to its proinflammatory cytokine activity, vasodilatory and antioxidant properties, as well as apoptotic activity of cervical and myometrial cells. The anti-inflammatory properties of RI may play a key role in reproductive tissue remodeling through recruitment of immune cells^39,40^ and regulation of subcellular signaling pathways, resulting in decreased cell proliferation and increased apoptosis. Such activity has been reported in human lung and skin cell study and is related to an increase in reductive versus oxidative stress.^41^

Polyphenols can also affect enzymatic and signaling systems, which are involved in inflammatory processes^42^ and can regulate various molecular targets.^43^ Antioxidants in RI likely cause death to cervical and myometrial cells, promoting architectural change important in triggering parturition processes by promoting protein signaling pathways, which increase inflammation, inhibit cell growth, and trigger cell cycle arrest.^44^ Furthermore, tannins in RI likely promote an important shift in microflora during cervical and myometrial architectural changes related to parturition.^45^ However, the bioactivity of RI polyphenols depends on activity and pharmacokinetics,^38^ which were not examined in this research and need further investigation.

The ability of kaempferol to modulate signaling pathways related to cell apoptosis and the high bioavailability of quercetin and promotion of inflammatory gene expression are likely influential in the role of RI to promote parturition. Furthermore, quercetin has been found to interfere in development of the endometrium during the peri-implantation period^27^ as well as decrease cell proliferation and induce cell death.^28^ Uterine immune cells stimulate protein production at term, which promote these processes.^46^ However, greater intake of polyphenols, particularly flavonoids, is reported to exert an anti-inflammatory effect by targeting one of two pathways (*i.e.*, arachidonic acid [AA]-dependent pathway and AA-independent pathway). High doses of RI may stimulate select AA-dependent proteins such as lipoxygenase (LOX), which is known to produce leukotrienes and other proinflammatory cytokines.^38,47^ This LOX pathway may also be key in determining the synthesis of prostaglandin (PG), which is crucial in cervical ripening and stimulation of uterine contractions. While some polyphenols prevent PG synthesis,^48^ findings from this research also suggest such inhibition is dose-dependent and targets an alternate pathway. Additionally, some polyphenols such as quercetin and catechins have been found to both enhance and inhibit production of select cytokines.^49^ Higher polyphenol doses of RI may disrupt the balance between pro- and anti-inflammatory cytokine production^48^ stimulating earlier parturition. Finally, the isoflavone genistein may also have been influential in decreasing the length of gestation, as chronic preconception exposure has been demonstrated to decrease the length of gestation in a mouse model. This difference has been attributed to estrogen receptor overexpression.^50^

RI also appears to have a physiologically active function in complementing oxytocin-induced contractions, with flavonoids effecting vasodilation activity.^51^ Research in murine models found that RI has a variable effect on uterine contractility, depending on preexisting oxytocin-induced contractions, herbal preparation, and pregnancy status.^17^ Similarly, application of RI to strips of animal and human uteri *in vitro* in nonpregnant states demonstrated mixed results,^15,16^ although there was evidence for use as a uterotonic^18^ demonstrating a physiologically active function in complementing oxytocin-induced contractions. RI extract appears to contain active constituents with muscle stimulant and spasmolytic activity, which relax isolated tissues,^52^ and researchers have suggested that there are at least two RI extract compounds with relaxant activity.^53^ Such activity may promote coordinated uterine contractions and dilatation of the cervix through effect on the lower uterine segment.^54^ Such activity is likely influential in cervical remodeling in late gestation. Taken together, these compounds may promote parturition; the effect of other RI phytochemicals is unknown. Polyphenols are known to have cytotoxic effects during cellular metabolism of compounds found in the extract,^27^ although it depends on the type of cells that are exposed.^55,56^ Little research has investigated the effects of chronic consumption of a polyphenol-rich diet during pregnancy on birth/fetal outcomes, and these findings further the understanding of the potential benefits and risk of RI ingestion. Because RI is rich in polyphenols, concerns have been raised about RI safety for human consumption,^57^ although studies have been limited by retrospective designs with small sample size and failure to verify dose and timing of RI use.

Dam gestational weight gain tended to decrease with increased RI dose when compared with controls, although the differences were not significant. Similarly, there were no significant differences between groups pertaining to food intake, but fluid consumption was significantly greater for those consuming high-dose RI when compared with controls (*P* < .05). This could be due to an increase in polyphenolics and flavonoids, which have demonstrated diuretic properties.^58^ Although this study did not measure urine excretion, there was no evidence of dehydration for any dam ingesting RI extract. Additional research is needed to ascertain the biological effects of RI.

The mean litter size was significantly smaller when comparing the RI high dose group with controls (*P* ≤ .05), and both the low- and high-dose groups delivered fewer viable pups compared with the control group. Higher dietary consumption of the polyphenols, particularly genistein in RI, may have decreased the number of implantations and surviving fetuses, which is consistent with the literature.^37,50,59^ The mechanism by which RI impacted litter size may have been influenced by quercetin and EGCG content. With high-dose RI, there were fewer viable and nonviable pups compared with controls; EGCG at high concentration has been found to increase pup loss in a rat model.^60^ Quercetin has been demonstrated to increase litter size.^61^ We hypothesize that quercetin at chronic high levels adversely effected fecundity in a dose-related manner, although additional research on its biological activity is needed. Finally, while litter size was not found related to additional pairings, additional research is yet needed. It has been hypothesized that high polyphenol ingestion, particularly quercetin, may contribute to early folliculogenesis as dams age.^61^

Finally, noteworthy findings in this research were the fact that eight dams, evenly spread across all groups, demonstrated weight gain after what appeared to be successful pairing. Weight gain increased the first week following pairing and then plateaued; dams were subsequently rebred. It is possible that there were litter losses, and despite no evidence of birth, fetuses may have been cannibalized. The breed of mouse may account for this finding and remote monitoring would have provided clarity. In addition, the large litter loss across groups, with low- and high-dose groups losing pups later, suggests chronic transplacental exposure may affect the well-being of offspring. This finding may have been compounded by the mouse strain used in this research as C57BL/6 have been reported to have considerable litter mortality rates.^62,63^

### Offspring characteristics and neurodevelopment

Assessing offspring characteristics demonstrated no differences in the appearance of hair, lower incisor eruption, or bilateral eye opening. There were, however, significant differences in weight, where those with high-dose maternal exposure had lower weights on PND4 and higher weights on PND5 (*P* ≤ .05). The higher weight in pups from high-dose groups was likely due to the fact that pups in smaller litters were able to access more food than pups from groups where there were high litter numbers.

Neurodevelopment was reflected in pup locomotive abilities including time to righting, orienting (geotaxis) response, cliff avoidance, and swimming development. None of the differences between groups were statistically significant. Assessments were not done daily for all measures, but rather occurred on select days from PND4 through PND12; daily measurements may have allowed for more nuanced differences. There was, however, a trend toward reduced neurodevelopmental behavior with exposure to RI, which became more marked with high-dose ingestion. On PND4 and PND6, high-dose RI exposure demonstrated a trend for longer time to righting; on PND5 and PND7, there was shorter time to righting; this finding is unexplained. Similarly, geotaxis reflex (PND8) did not significantly differ between groups, although there was a trend toward increased response time for RI-treated groups; cliff avoidance reflex time (PND10) decreased as the RI dose increased compared with placebo. Overall, differences in pup loss rates, offspring weights, and select neurodevelopment parameters give evidence of the metabolism and transfer of RI metabolites to fetal tissues, impacting developmental processes.

General body strength was reflected in swimming development at PND12 by observing swim direction, head position, line of swim, and use of head, paws, and tail movement. Scores (0–2) were assigned for each, and a total score was determined; no significant differences were demonstrated for each component or for the overall swim score between groups. There was, however, a trend toward lower hind leg position, tail movement, and swim line component scores, as well as in the overall total swim score with RI treatment. Development of swimming requires total coordination of muscles, with habituation increasing scores.^64^ This suggests that while physical maturation is influential, RI exposure *in utero* may result in diminished swimming skills further exacerbated by one-time testing (PND12). Research has demonstrated that diet has an impact on mice learning abilities,^65^ although it remains unknown how chronic transplacental exposure impacts long-term neurodevelopment, or how it might influence subsequent generations.

This study, which evaluated the effect of RI on select parameters in mice and their offspring when ingested throughout gestation, found evidence of RIs impact on gestational length, fluid intake during pregnancy, litter size and viability, as well as pup development. Findings suggest safety concerns at higher doses with the need for further research. Targeted *in vitro* and *in vivo* studies are yet needed to further specify the physiologically active function of RI and potential interactions with differing biochemical processes. Such studies should include mechanisms of action, varied routes of product delivery, effects on fertility, organogenesis, implantation and placentation, and the growth and neurological effects on maternal mice and pups.

### Limitations

There were several limitations in this study. First, the precise time of birth could not be determined as remote monitoring was unavailable. Cages were checked each morning to determine birth, as mice typically give birth during the night. Keeping mice in a metabolic cage and recording wastage would have been more precise in determining the intake of RI extract. Second, use of C57BL/6N Tac mice may have influenced findings, as they are not known to have good mothering behavior and the strain has been reported to have considerable litter mortality rates.^62,63^ Third, handling of pups before weaning may have been influential in findings as manipulation during the first and second weeks of postnatal development likely impacts a critical period related to environmentally induced changes in neurobiology.^66^ Fourth, necropsies were not performed, which would have been helpful in identifying offspring gender, as well as external, visceral, or skeletal malformations, and potential impact on dam reproductive capabilities. Finally, the quality of the RI raw product cannot be assured as an independent laboratory did not conduct analysis of the plant material to identify chemical content, as well as unwanted adulterants. Industry standard is to do so with liquid chromatography-mass spectrometry (LCMS) methods to assess purity, bioavailability, toxicity, metabolism, and molecular target profiling.^67^

In summary, RI has long been reported to shorten gestation, stimulate labor, and improve pregnancy outcomes despite relatively little scientific evidence. Findings from this research demonstrate effect on gestational changes, which support parturition processes, fecundity, offspring characteristics, and neurodevelopment. Findings underscore the need for further research regarding dose-related impact, and the need to determine the chemical composition of the extract (*i.e.*, total phenols and isoflavones, anthocyanins, tannins).

There has been limited drug development to target parturition-related problems,^7^ and RI may have therapeutic efficacy. The development of new products from medicinal plants is encouraged as the demand for botanically derived products grows.^68,69^ Because of the popularity of botanicals during pregnancy—particularly RI—there is continued need to fully understand its impact on gestation and offspring.
